# Cost-effectiveness analysis of gemtuzumab ozogamicin for the treatment of de novo CD33-positive Acute Myeloid Leukaemia (AML) in Italy

**DOI:** 10.1186/s12913-023-09054-x

**Published:** 2023-01-16

**Authors:** Roberto Cairoli, Gianluca Furneri, Roberto Di Virgilio, Barbara Veggia, Felicetto Ferrara

**Affiliations:** 1 Department of Haematology, ASST Grande Ospedale Metropolitano Niguarda, Milan, Italy; 2 PharmaLex Italy S.p.A., Milan, Italy; 3 Pfizer Italia, Rome, Italy; 4 Department of Haematology, Ospedale “Antonio Cardarelli”, Naples, Italy

**Keywords:** Gemtuzumab ozogamicin, Acute myeloid leukemia, ALFA-0701 study, Cost-effectiveness, Italy

## Abstract

**Background:**

Based on the results from the ALFA-0701 study, gemtuzumab ozogamicin (GO) has been approved by the European Medicine Agency and by the Italian Drug Agency for the first line treatment of de novo acute-myeloid leukemia (AML). In this analysis, we assessed the cost-effectiveness of GO in combination with daunorubicin and cytarabine (DA), vs DA alone, adopting the perspective of the Italian National Health Service.

**Methods:**

For this analysis, a cohort state transition model was developed. The model was designed to capture health states and events that occur throughout the entire disease course and that impact costs and outcomes. The ALFA-0701 study was the main source of clinical data for this analysis. In the model, patients had the same baseline characteristics and experienced the same clinical improvements as in the ALFA-0701 study. Economic data (resource consumption and unit costs) were adapted to reflect expenditure for the Italian National Health Service. Utilities per health state and disutilities due to adverse events were based on the literature and on the general population for those functionally cured. A lifetime horizon was adopted, with both costs and outcome being discounted of 3.0%, annually. Deterministic and probabilistic sensitivity analyses were conducted to assess the robustness of results.

**Results:**

In the base case (lifetime horizon; primary source of data: study ALFA-0701; perspective: Italian National Health Service; discount rate on costs and outcomes: 3.0%), GO + DA was more effective DA both in terms of life-year (LY) survival (6.42 LY vs 5.75 LY, respectively) and quality-of-life adjusted survival (4.69 QALY vs 4.19 QALY, respectively). The overall costs were almost similar in the two groups (slightly lower with GO + DA than with DA; €162,424 and €162,708, respectively). The use of GO increased the costs of drug therapy but saved costs of relapse and costs associated with transplantation (HSCT).

**Conclusions:**

If results of the ALFA-0701 study are applied to the Italian healthcare environment, then gemtuzumab ozogamicin, in combination with daunorubicin and cytarabine, would clinical outcomes and reduce lifetime costs, compared with daunorubicin and cytarabine alone for the first line treatment of de novo AML.

**Trial registration:**

Not applicable.

**Supplementary Information:**

The online version contains supplementary material available at 10.1186/s12913-023-09054-x.

## Background

Acute myeloid leukemia (AML) is the most common type of acute leukemia in adults [1]. In Italy, AML incidence is about 3.5 patients per 100,000 inhabitants/year [2], but being a typical disease of elderly people, it can also reach about 10 cases per 100,000 inhabitants/year in the population over 65 years old (70% of total cases) [3]. Advances in the treatment of AML have resulted in substantially improved rates of complete remission (CR) achievement [4]. Approximately 60% to 70% of adults with AML are expected to achieve CR following appropriate induction therapy. More than 25% of adults with AML (about 45% of those who achieve CR) survive 5 or more years [5]. However, remission rates in adults with AML are inversely related to age, pre-existing clonal hematopoietic disorders such as myelodysplastic syndrome and certain somatic genetic abnormalities which also confer a worse prognosis [1].

The standard induction therapy for newly diagnosed AML patients who are fit for intensive chemotherapy consists of the association of anthracyclines, such as daunorubicin, on each of the first 3 days, and cytarabine (AraC) in continuous infusion for 7 days [6]. Post-remission therapeutic strategies include intensive chemotherapy and chemotherapy at higher doses AraC followed by allogeneic transplantation or, in patients with favorable prognostic factors, autologous transplantation or further high dose AraC courses [7]. More recently, in 2020, gemtuzumab ozogamicin (GO) was included in the ESMO guidelines in addition to the standard therapy both for the induction and consolidation treatment [8] in patients with favorable or intermediate cytogenetics.

Gemtuzumab ozogamicin (Mylotarg; Pfizer Inc.) is an anti-CD33 antibody conjugate covalently linked to the cytotoxic agent N-acetyl gamma calicheamicin. Binding of the anti-CD33 antibody portion of Mylotarg with the CD33 antigen, expressed on the surface of leukemic blasts, results in the formation of a complex that is internalized. Upon internalization, the calicheamicin derivative is released inside the lysosomes of the myeloid cell resulting in DNA double strand breaks and cell death [9].

In 2018, Mylotarg was approved by the European Medicines Agency (EMA) for the treatment of patients aged 15 years and above affected by de novo CD33-positive AML, except acute promyelocytic leukemia (APL), in combination with daunorubicin (DNR) and cytarabine (AraC) [10]. In 2019, Mylotarg was approved for reimbursement by the Italian Drug Agency (AIFA).

GO market authorization is based on the ALFA-0701 study, a pivotal trial comparing GO plus standard therapy DA (daunorubicin and cytarabine) against daunorubicin and cytarabine alone in patients aged 50 to 70 years with previously untreated, de novo AML [11].

The objective of this analysis is to evaluate cost-effectiveness of GO, in combination with daunorubicin and cytarabine (DA), vs DA alone, for the first line treatment of de novo AML, adopting the perspective of the Italian National Health Service (NHS). Specifically, the analysis estimates costs and outcomes of treating Italian AML patients with these two alternative therapeutic regimens, assuming they would follow the treatment protocol adopted in the ALFA-0701 study [11].

## Methods

### Model design

**Fig. 1:**
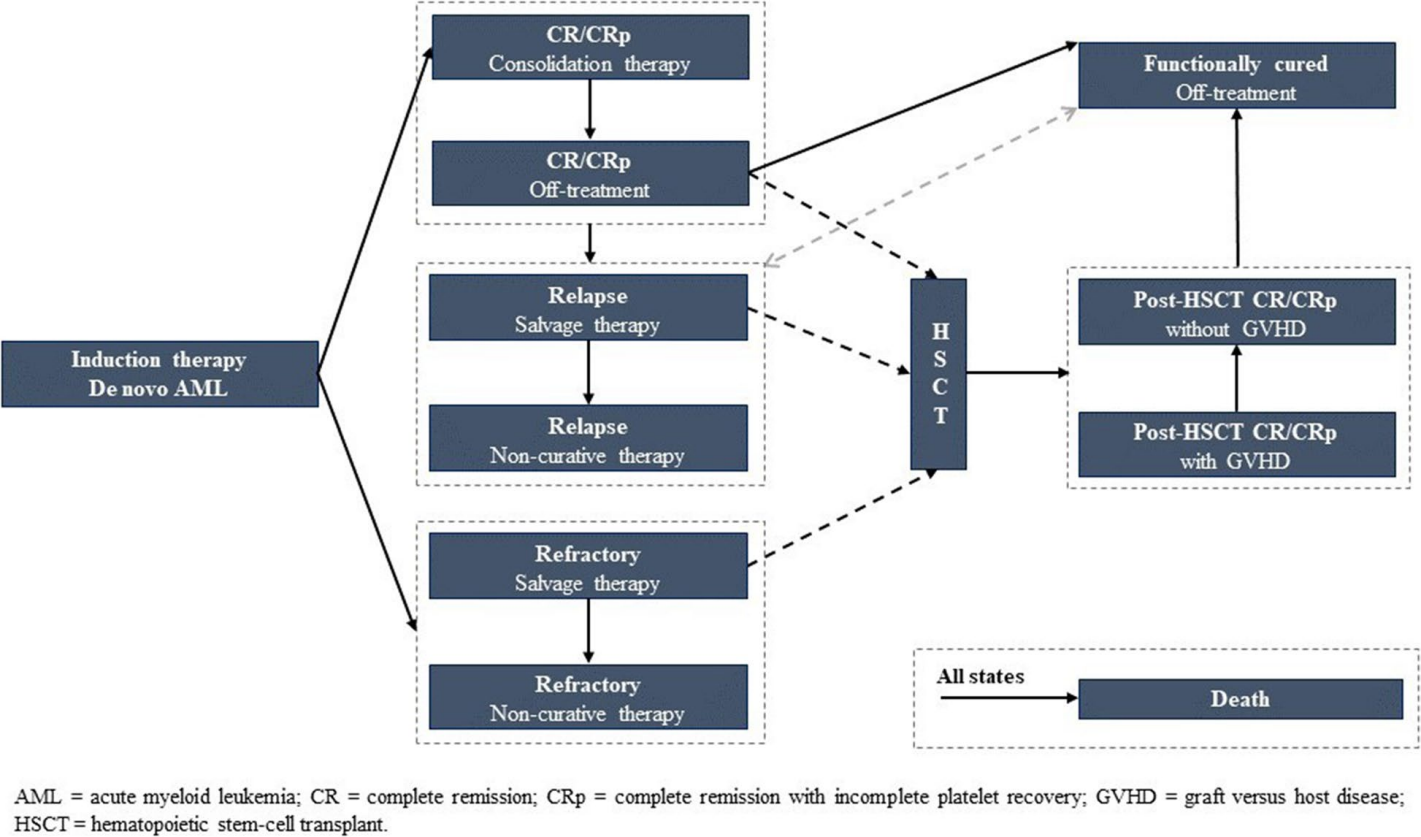
Model structure

The assessment of costs, clinical outcomes and survival adjusted for quality of life associated with GO + DA compared to DA alone was carried out through a cost-effectiveness analysis. For this purpose, a cohort state transition model was developed. This model was an upgrade of the partitioned survival models that are traditionally used in oncology. The model structure was designed to capture health states and events that occur throughout the entire disease course and that impact costs and outcomes. In total, 12 health states were identified to simulate the disease trajectory of de novo AML patients during their diagnostic and treatment pathway (Fig. 1).

Simulation starts with de novo AML patients receiving their systemic chemotherapy (either GO + DA, or DA). Patients can receive either one or two induction cycles, depending on their initial treatment response. After induction completion, patients will be in one of the following health states: i) complete remission (CR); ii) complete remission with incomplete platelet recovery (CRp); iii) induction failure (refractory patients, non-responders). Patients achieving CR or CRp start consolidation therapy, consisting in two additional treatment cycles. After consolidation therapy, a certain proportion of patients would be eligible for hematopoietic stem-cell transplant (HSCT). Patients with initial CR or CRp who relapse receive a second-line treatment, which is still an active treatment (savage chemotherapy), in 60% of cases [12], or best-supportive care (non-curative treatment) in 40% of cases [12]. The overall survival of these patients was estimated from literature data [13], as this was not directly captured in the ALFA-0701 registrational trial.

A certain proportion of patients, who respond to the study treatment or to savage chemotherapy, will receive HSCT. Therefore, three different patient subgroups are candidates for HSCT: i) patients achieving complete remission (CR or CRp) after first line therapy (group A); ii) patients initially achieving complete remission with first line induction therapy, but relapsing afterwards, and then achieving again remission after second line therapy (group B); iii) patients not responding to first line induction therapy, but achieving remission after second line therapy (group C).

In the model, patients who maintain remission for at least 60 months are considered functionally cured. This assumption is primarily supported by the evidence from the ALFA-0701 study [11], showing that RFS and OS Kaplan–Meier curves had a plateau from approximately 48 months to 60 months, meaning that patients were disease-free at that time; this was also confirmed by a panel of UK experts [12]. In light of such assumption, these patients are assumed to have same overall survival as the general population. Similarly, a certain proportion of patients receiving HSCT are functionally cured and have long-term survival [11].

In clinical practice, non-curative treatments are initiated when other treatment options are not available or not recommended [14, 15]. These treatments are supposed to continue until patients die. The model uses the restricted mean survival time (RMST) of relapsing and refractory patients in the ALFA-0701 study [11] to estimate duration and costs of this non-curative therapy.

In this model, patients are observed over a maximum time horizon of 40 years (equivalent to a lifetime horizon). A discount rate of 3.0% was applied to both costs and outcomes [16]. The Italian National Health Service (NHS) perspective was adopted; therefore, only direct medical costs were considered in the analysis.

### Clinical inputs

#### Characteristics of patients at baseline

The study population is a hypothetical cohort of patients with previously untreated de novo AML who are eligible to receive an intensive chemotherapy regimen. In this analysis, patients have the same baseline characteristics as in the ALFA-0701 study [11]: mean age of 61.5 years (standard deviation -SD- 5.24), average Body Surface Area (BSA) of 1.83 m^2^ (SD 0.20), average weight of 74.4 kg (SD 15.19) and 50.55% proportion of women.

Age and distribution by gender were used to calculate annual mortality rates, using Italian mortality tables [17]. Body surface area and weight data were used to calculate treatment doses and associated costs.

#### Treatment alternatives

The interventions included in the model and the treatment regimens are presented in Table 1. GO is given in combination with DA, which is the standard therapy used in clinical practice.

**Table 1:** Treatment regimens of study interventions [Source: [11]]

Treatment	Treatment regimens
GO + DA (intervention)	**Induction, course 1**:
	GO = 3 mg/m^2^ per day (max = 5 mg), on days 1–3 (2-h IV infusion)
	DNR = 60 mg/m^2^ per day, on days 1–3 (30-min IV infusion)
	AraC = 200 mg/m^2^ per day, on days 1–7 (continuous IV infusion)
	**Induction, course 2**:
	DNR = 35 mg/m^2^ per day, on days 1–3 (30-min IV infusion)
	AraC = 1,000 mg/m^2^ per 12 h, on days 1–3 (12-h IV infusion)
	**Consolidation, course 1**^a^:
	GO = 3 mg/m^2^ per day (maximum = 5 mg), on day 1 (2-h IV infusion)
	DNR = 60 mg/m^2^ per day, on day 1 (30-min IV infusion)
	AraC = 1,000 mg/m^2^ per 12 h, on days 1–4 (2-h IV infusion)
	**Consolidation, course 2**^a^:
	GO = 3 mg/m^2^ per day (maximum = 5 mg), on day 1 (2-h IV infusion)
	DNR = 60 mg/m^2^ per day, on days 1 and 2 (30-min IV infusion)
	AraC = 1,000 mg/m^2^ per 12 h, on days 1–4 (2-h IV infusion)
DA (comparator)	**Induction, course 1**:
	DNR = 60 mg/m^2^ per day, on days 1–3 (30-min IV infusion)
	AraC = 200 mg/m^2^ per day, on days 1–7 (continuous IV infusion)
	**Induction, course 2**:
	DNR = 35 mg/m^2^ per day, on days 1–3 (30-min IV infusion)
	AraC = 1,000 mg/m^2^ per 12 h, on days 1–3 (2-h IV infusion)
	**Consolidation, course 1**^a^:
	DNR = 60 mg/m^2^ per day, on day 1 (30-min IV infusion)
	AraC = 1,000 mg/m^2^ per 12 h, on days 1–4 (2-h IV infusion)
	**Consolidation, course 2**^a^:
	DNR = 60 mg/m^2^ per day, on day 1 and 2 (30-min IV infusion)
	AraC = 1,000 mg/m^2^ per 12 h, on days 1–4 (2-h IV infusion)

*AraC =* cytarabine, *CR* = complete remission, *CRp =* complete remission with incomplete platelet recovery, *DA* = daunorubicin and cytarabine, *DNR =* daunorubicin, GO = gemtuzumab ozogamicin, IV = intravenous

^a^Given only to those patients who attained CR or CRp following induction therapy

#### Treatment effectiveness

Five key clinical parameters of effectiveness and safety were used in the model: i) treatment response (CR or CRp); ii) event-free survival (EFS); iii) overall survival (OS); iv) hematopoietic stem-cell transplant (HSCT) probability; v) frequency of adverse events.

**Fig. 2:**
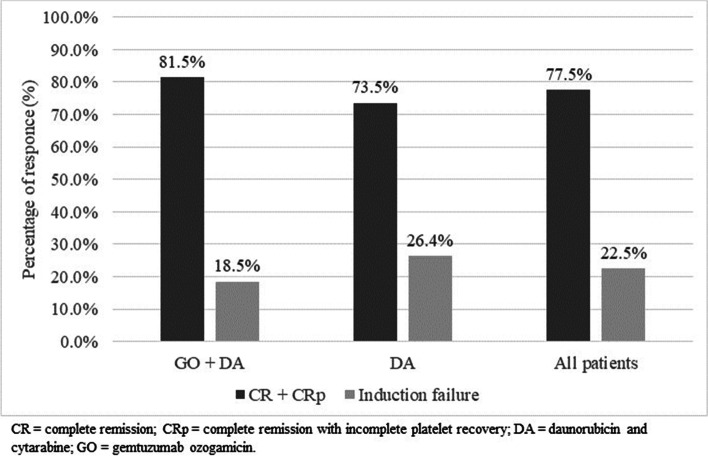
Response rates after induction treatment [18]

Treatment response after first line induction, intended as complete remission (CR) or complete remission with incomplete platelet recovery (CRp), was collected from the ALFA-0701 study (Fig. 2). Patients not achieving CR or CRp were classified as non-responders. According to the ALFA-0701, a larger proportion of patients achieved remission after induction (82% vs 74%) if treated with GO in combination with DA compared to DA alone [11].

**Fig. 3:**
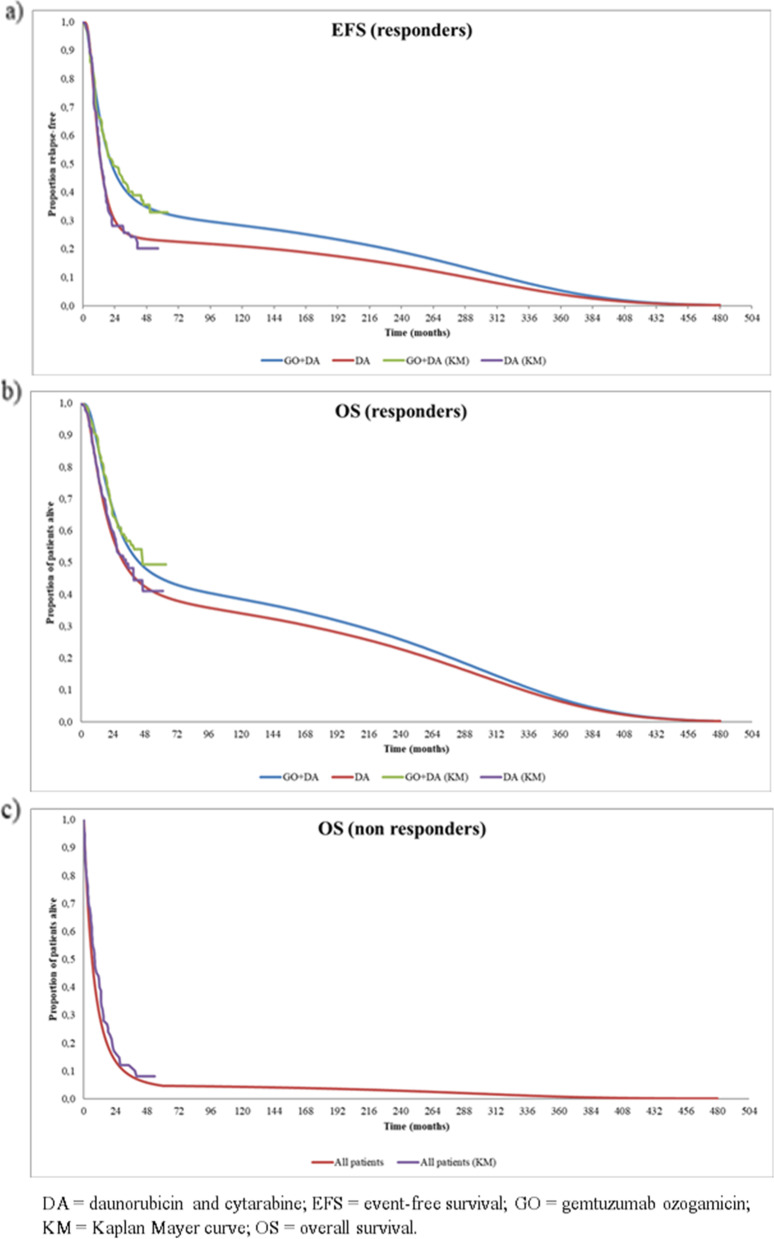
**a** EFS in induction responders; **b** OS in induction responders; **c** OS in induction non-responders

EFS and OS functions were estimated from the ALFA-0701 study [11]. As expected, both parameters strongly depended on response status. Therefore, EFS and OS were estimated separately for responders (achieving CR or CRp) and non-responders (induction failure). Figure 3 shows EFS (a) and OS (b) curves for responders, by treatment. For both parameters, long-term extrapolation was optimized through log-normal parametrization. For non-responders, OS was assumed not dependent on received treatment; therefore, OS of non-responders was not stratified by treatment. A Gompertz function was used to extrapolate long-term OS of these patients (Fig. 3c).

As already described in the model design section, three different patient subgroups are candidates for HSCT in the model: i) patients achieving complete remission (CR or CRp) after first line therapy (group A); ii) patients initially achieving complete remission with first line induction therapy, but relapsing at a certain point, who achieved again remission after second line therapy (group B); iii) patients who did not respond to first line induction therapy (failures), who achieved remission after second line therapy (group C). All probabilities of receiving HSCT were retrieved from the ALFA-0701 study [11]. For patients responding to the induction therapy (group A), HSCT probability was 8.6%. For patients initially responding to induction therapy, but then relapsing (group B), HSCT probabilities were found to be time-dependent, as shown in Table 2. For patients not responding to induction therapy, but in remission with second line therapy (group C) HSCT probability was 18.0%. In the ALFA-0701 study [11], almost all HSCTs were allogenic; therefore, the model assumption was that 100% of HSCTs were allogenic.

**Table 2:** Annual probability of HSCT for relapsed patients [Source: [11]]

Probability of HSCT	GO + DA	DA	Pooled^a^
Year 1, %	9.1	14.0	11.4
Year 2, %	7.3	14.0	10.5
Year 3, %	0.9	3.0	1.9
Year 4, %	2.7	1.0	1.9
Year 5, %	0.0	0.0	0.0

*DA* = daunorubicin and cytarabine, *GO=* gemtuzumab ozogamicin, *HSCT* = hematopoietic stem-cell transplant

^a^Pooled data were calculated from the individual treatment-arm data reported in the ALFA-0701 study [8]

The model also estimates the costs of treatment-related adverse events (AE) management and the disutilities associated to such events. Only grade 3–4 AEs occurring in at least 1% of patients in the ALFA-0701 study [11], were considered, assuming that only these events would have a non-negligible impact on patients’ costs and quality of life (Table 3).

**Table 3:** Frequency of Adverse Events for First-Line AML Therapies [Source: [11]]

Adverse event, n (%)	GO + DA (N = 131)	DA (N = 137)
Skin toxicity	14 (10.7)	23 (16.8)
Mucosal toxicity	21 (16.0)	9 (6.6)
Pain	19 (14.5)	5 (3.6)
Nausea, vomiting e diarrhea	22 (16.8)	14 (10.2)
Pulmonary toxicity	17 (13.0)	19 (13.9)
Cardiac rhythm disorder	5 (3.8)	4 (2.9)
Other cardiac toxicity	6 (4.6)	5 (3.6)
Central neurological toxicity	8 (6.1)	4 (2.9)
Peripheral neurological toxicity	4 (3.1)	2 (1.5)
Infections	102 (77.9)	106 (77.4)
Hemorrhage	30 (22.9)	13 (9.5)
Veno-occlusive disease	5 (3.8)	2 (1.5)

*AML* = acute myeloid leukemia, *DA =* daunorubicin and cytarabine, *GO =* gemtuzumab ozogamicin

### Utility inputs

Health related quality of life (HRQoL) data were not collected during the ALFA-0701 study. Therefore, utility data by different health states were extrapolated from literature review [13], and from an elicitation study conducted by Pfizer [19]. According to NICE recommendations [20], utility data were estimated through EQ-5D (Table 4).

**Table 4:** Utilities associated with the health states of the model [Source: in the notes]

Health states	EQ-5D (Default)^a^
Chemotherapy treatment^b^	0.6574
Consolidation therapy	0.6574^c^
HSCT procedure	0.6574^c^
GVHD (post HSCT)	0.6700^c^
CR o CRp	0.7400
Relapse	0.5680
Refractory	0.5680^d^
Functionnaly cured	0.8199^e^
Dead	0.0000

*CR* = complete remission, *CRp* = complete remission with incomplete platelet recovery, *EQ-5D* = European quality of life 5 dimensions, *GVHD* = graft versus host disease, *HSCT =* hematopoietic stem-cell transplant

^a^Values from TA399 of NICE [21], using the mapping algorithm by McKenzie and Van der Pol (2009) [22]

^b^Includes patients who are receiving induction or salvage chemotherapy

^c^Value from Kurosawa et al. (2016) [23]

^d^Assumed equal to relapse

^e^Calculated using baseline patient characteristics for all patients in the ALFA-0701 study [11]

### Cost inputs

Consistently with the adopted perspective, the following direct healthcare costs related to the pharmacological treatment and healthcare management of de novo patients with AML were identified, measured and quantified: i) cost of first-line treatment; ii) cost of adverse events associated with first-line treatment; iii) cost of subsequent lines of treatment (including non-curative therapy, and end-of-life care); iv) cost of allogeneic hematopoietic cell transplantation (HSCT); v) monitoring and follow-up costs associated with health status; vi) cost of end of life care.

The costs of first-line drug therapy were calculated considering the specific treatment regimens for each drug as specified in study ALFA-0701 [11]. The average cost of the drug per dose administered was calculated assuming that there is no drug wastage and using the minimum price per milligram. First-line treatments are administered on an inpatient basis. Table 7 (supplementary material) illustrates the unit costs for first-line treatments.

In the analyses the costs of grade 3–4 treatment-related adverse events were considered. The incidence of events was estimated from the ALFA-0701 study [11]. To each grade 3–4 adverse event, a cost reflecting the expenditure for the Italian SSN was assigned. For veno-occlusive disease (VOD), cost was calculated summing up the cost VOD diagnosis (DH 207;208 [24]) and the expense for drug treatment. In absence of direct information from the study ALFA-0701 on VOD treatment schedule, the AML 17 study protocol [25] was used to inform costs; the protocol recommended administering a total of 10 mg/kg of defibrotide every day for 7 days [25]. Considering a unit cost of defibrotide (200 mg) equal to €474.09 [26], the cost of the pharmacological treatment was equal to €12,345.33 per episode (~ €2.37/mg x 10 mg/kg × 74,40 kg × 7 days).

Relapsed and refractory patients who are deemed functionally eligible can receive rescue chemotherapy [27]. Standard second-line rescue therapy includes the use of fludarabine, cytarabine, granulocyte colony stimulating factor (G-CSF) and fludarabine, cytarabine, G-CSF and idarubicin (FLAG-Ida) [27]. It was assumed that all patients receiving salvage therapy in the model receive FLAG-Ida, as validated by clinical experts [28]. Patients generally receive one or two courses of FLAG-Ida. In the absence of available data, it was assumed that patients received an average of 1.5 cycles. Rescue therapy is administered in a hospital setting, so the cost of administration is incorporated into the cost of hospitalization. Relapsed and refractory patients who are deemed ineligible to receive rescue therapy, instead receive non-curative therapies (including best-supportive care) and palliative care [14, 15]. According to the opinion of experts, the three most used therapies in this area are: i) hydroxycarbamide; ii) low dose cytarabine; iii) azacytidine. In the model it was assumed that these therapies are used in a 40:40:20 ratio, respectively [28]. Patients who have received salvage therapy and have not received a transplant (HSCT) will only receive best-supportive care. Non-curative therapies are continued until there is no clinical/symptomatic benefit and are assumed to continue until terminal care begins [14, 15]. To calculate the duration of non-curative therapies before the cost of end-of-life care (applied for 2 cycles), the model uses the RMST (narrow mean survival time) estimates from ALFA-0701 [11] for relapsed and refractory patients. The base case used pooled estimates of RMSTs lasting 10.07 months for patients with new relapse and 7.95 months for refractory patients.

Patients who respond to treatment (first or second line) are eligible (if clinically and biologically fit) to receive hematopoietic stem-cell transplantation (HSCT). In the ALFA-0701 study [11] nearly all transplants were allogeneic. Therefore, HSCT in the model were considered allogeneic, based on expert opinion [28]. The unit cost of HSCT was obtained from Lucioni study (2015) [29] and inflated to the costs of 2021 [30]. The cost was split between the cost of the HSCT procedure, which includes the post-transplant recovery period, and the costs associated with follow-up in the two years following HSCT. The inflated costs of HSCT used in the model are shown in Table 7 (supplementary material). No additional transplant-related costs were applied after the 2-year period following the hematopoietic stem cell transplant. The model also considers the complications of acute and chronic transplant-related acute rejection disease (GVHD).

The direct costs associated with the management of patients with AML which are not specifically related to systemic therapy were: hospitalizations; specialistic visits; diagnostic tests; support therapy; transfusions. Resources and costs were calculated for each treatment phase within the health status and costs were applied in each cycle of model (Table 7, supplementary material).

In addition to the costs of first-line treatment, management of adverse events, subsequent lines, monitoring, and transplantation (HSCT), the costs related to patient management in the last 8 weeks of life were considered in the model. The cost of end-of-life care was calculated considering the value of a 10-day hospitalization immediately before death (€780/day), equal to €7,901.40 (Table 7, supplementary material). These costs were calculated by processing data from the Lucioni study [29], re-evaluated to 2021 [30].

### Sensitivity analysis

Deterministic (one-way) and probabilistic sensitivity analyses were carried out to identify the input values with the largest effect on incremental cost-effectiveness ratio (ICER).

For the deterministic sensitivity analysis, the baseline value of each parameter was modified to the upper and lower limits of a variation of ± 10%. It was decided to also vary the economic data by ± 10% (e.g., HSCT costs), although the latter were not (plausibly) affected by a high level of uncertainty.

Probabilistic sensitivity analysis was also performed, simultaneously and randomly varying the values of all model parameters (1,000 replications). For the probabilistic analysis, the following probability distributions were used: beta for probabilities, proportions, incidences, utilities, and rates; normal for costs.

Finally, additional scenario analyses were carried out to test robustness of the analysis. Cost-effectiveness of GO + DA vs DA was assessed by: i) cytogenetic profile (all population or favorable and intermediate risk) as per ELN2017 definitions; ii) ± 5% HSCT rate in patients achieving remission after study treatment; iii) ± 5% HSCT rate in patients who were refractory to the study treatment.

## Results

### Base-case analysis

In the base case (time horizon: 40 years; primary source of data: study ALFA-0701; perspective: SSN; discount rate on costs and outcomes: 3.0%), GO + DA was more effective than the comparator (DA) both in terms of survival (6.42 LY vs 5.75 LY, respectively) and quality-of-life adjusted survival (4.69 QALY vs 4.19 QALY, respectively; Table 5).

**Table 5:** Results of cost-effectiveness analysis

Parameter	GO + DA (a)	DA (b)	Difference (a-b)	ICER, a vs b (€)
Total costs (€)	162,424	162,708	-285	-
Life Years (LYs)	6.42	5.75	0.67	-425
QALYs	4.69	4.19	0.50	-568

*DA =* daunorubicin and cytarabine, *GO =* gemtuzumab ozogamicin, *ICER =* incremental cost-effectiveness ratio, *LY =* life year, *QALY* = quality adjusted life year

The overall costs were almost similar in the two groups (slightly lower with GO + DA than with DA; €162,424 and €162,708, respectively). The use of GO causes an increase in the costs of drug therapy, since it is an add-on therapy, but allows savings in terms of cost of relapse and costs associated with transplantation (HSCT).

Considering these results, GO + DA is formally dominant compared to DA, with an incremental cost-effectiveness ratio (ICER) of -€425 per year of life earned, and -€568 per QALY earned. Basically, GO + DA improves outcomes compared to DA, at similar costs for the SSN.

### Sensitivity analysis

Both one-way deterministic and probabilistic sensitivity analyses confirmed the robustness and reliability of base-case results.

**Fig. 4:**
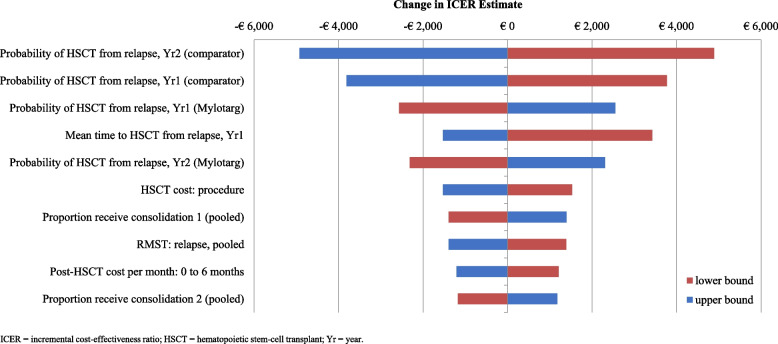
Results of one-way deterministic sensitivity analysis

The results of one-way deterministic analysis are summarized in Fig. 4, that illustrates the 10 parameters with the greatest effect on ICER. The variability of the ICER was modest (minimum ICER: -€5,494/QALY earned; maximum ICER: €4,319/QALY earned).

**Fig. 5:**
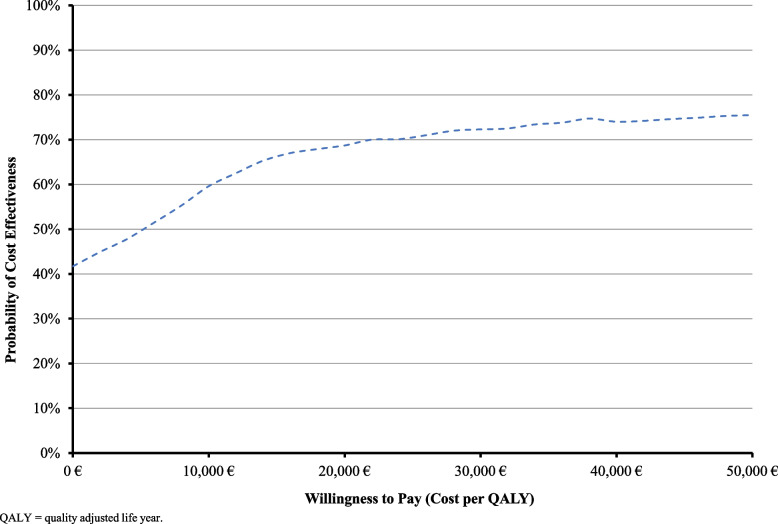
Results of probabilistic sensitivity analysis: cost effectiveness acceptability curve

The results of the probabilistic sensitivity analysis are shown in Fig. 5. The acceptability curve showed that when the willingness to pay (WTP) is equal to €50,000 per QALY gained, GO + DA has a probability of 76% to be cost-effective compared to DA.

In the favorable and intermediate cytogenetic risk subgroup, GO + DA was dominant vs DA, with an ICER of -€888 per QALY gained (Table 6). As expected, in this setting both cost difference and QALY difference were more favourable than in the base-case, in line with the improved survival observed in this subgroup.

**Table 6:** Results of the scenario analysis (favorable and intermediate cytogenetic risk subgroup)

Parameter	GO + DA (a)	DA (b)	Difference (a-b)	ICER, a vs b (€)
Total costs (€)	166,615	167,414	-799	-
Life Years (LYs)	7.61	6.41	1.20	-664
QALYs	5.57	4.67	0.90	-888

*DA* = daunorubicin and cytarabine, *GO* = gemtuzumab ozogamicin. *ICER* = incremental cost-effectiveness ratio, *LY =* life year, *QALY* = quality adjusted life year

Finally, modification of HSCT rates in the analysis was found to have a minor impact on ICER: GO + DA remains dominant compared to DA modifying the HSCT rates.

## Discussion

This cost-effectiveness analysis shows that, at the current price agreed with the Italian NHS, the addition of gemtuzumab ozogamicin (GO) to conventional induction chemotherapy regimen based on daunorubicin and cytarabine (DA), is dominant vs conventional induction chemotherapy alone. In the analysis, the incremental investments required to add GO to DA are offset by the reduction of relapse costs and by the lower incidence of hematopoietic stem-cell transplants (HSCT), as more patients in the GO + DA group achieved complete remission and functional cure without further therapy, compared to DA alone.

Despite different acquisition costs and settings, the results of this Italian analysis are in line with those from other published economic analyses in other countries, UK [31], Spain [32], Portugal [33], which used a similar model to conduct the evaluation. Overall, the fact that GO has been reimbursed in most European countries and has been recommended by several health technology assessment bodies worldwide, for example National Institute for Health and Clinical Excellence (NICE) in England [34]; Scottish Medicines Consortium (SMC) in Scotland [35]; Canadian Agency for Drugs and Technologies in Health (CADTH) in Canada [36], proves that GO has been extensively acknowledged as a cost-effective option in previously untreated, de novo CD33 AML.

From an economic point of view, the major strength of GO is that it is administered for a fixed number of cycles (unlike many other novel treatments in hematological malignancies) and can be timely stopped after one or two cycles of induction, if the patient does not experience hematological remission. In this way, the overall drug investment is limited, and it is continued only after early confirmation of response/remission. On the other side, the achievement of higher rates of complete remission (CR or CRp) with GO + DA, observed in the ALFA-0701 study, is an important clinical milestone as it increases patient chances to remain disease free for long, and potentially be cured [11].

Like most cost-effectiveness models, the present analysis has some degree of uncertainty that should be carefully evaluated. First, the model has a relatively high level of complexity: it is a cohort state-transition model, with 12 health-states, while in many models used in cancer there are only 3 health-states: pre-progression, post-progression, and death. Despite someone could argue that such complexity makes the model too sophisticated, and requires many assumptions, we still believe that such increase in complexity clearly reflects the complexity of AML management, therefore it represents a key-strength and not a point of weakness; the fact NICE and other HTA agencies accepted the model structure means that a less complex approach, based on a simpler partitioned survival model, would have not been efficient in capturing the different phases of the disease. Also, model complexity is somehow offset by the fact that most clinical inputs come from the ALFA-0701 registrational study, which informed on superiority of GO + DA vs DA considered an appropriate comparator therapy in untreated AML patients not harboring mutations. Therefore, a cost-effectiveness analysis was conducted without using any external control, or indirect treatment comparison method.

As previously mentioned, a complex model requires several assumptions that were validated by expert opinion, in absence of a more robust clinical source. In particular: i) putting the threshold of functional cure at 5 years; ii) setting the proportion of relapsed and refractory patients receiving salvage therapy at 60%; Regarding i), we believe with authors of the other cost-effectiveness analysis that the assumption is conservative, since reducing this time would favor GO + DA, while increasing it would not be realistic. Second, modifying the proportion of relapsed and refractory patients receiving salvage therapy from this base-case estimate of 60% does not have a large impact on ICER (40% €513 – 80% -€1,650).

Another limitation of the analysis is about model utilities: i) health-related quality-of-life data were not collected in the ALFA-0701 study, therefore, the utility estimates were obtained from other sources; ii) utilities were not adapted to the Italian patients. We are aware of these two limitations; however, given the favorable ICER of the base-case analysis, even a less favorable utility assessment is not expected to change the direction of the analysis and the final recommendations.

In certain countries, like England, GO was recommended and reimbursed for patients when either the cytogenetic test confirming that the disease has favorable, intermediate, or unknown cytogenetics (or when their cytogenetic test results are not yet available) [34], because it was seen from subgroup analysis that patients with favorable or intermediate cytogenetic risk had a significantly longer EFS in the GO arm versus the control arm (HR: 0.46, p < 0.0001), which was not observed in patients with poor cytogenetic risk (HR: 1.11, p = 0.72) [37]. However, this reimbursement restriction is not applied in Italy [38]; indeed, results of the cost-effectiveness analysis improve in the subgroup with favorable or intermediate cytogenetic risk; however, GO + DA is still dominant vs DA, when the ITT population of the ALFA study is considered. Therefore, we could conclude that the Italian Drug Agency preferred a broad reimbursement (as per EMA label), thus giving physicians the option of assessing the opportunity of treatment in any AML patient.

Also, we acknowledge that the present analysis was run under the assumption that patients would follow the treatment protocol adopted in the ALFA-0701 study. Indeed, several adjustments to this protocol have been observed in clinical practice, regarding, for example, modification of the chemotherapy treatment schedule, modification of the chemotherapy regimen, GO dosage, or eligibility criteria for HSCT. Of course, we were not in a condition to simulate all the possible treatment adjustments; however, results of sensitivity analyses and scenario analyses confirm that ICER is not subject to significant variability when underlying assumptions are modified.

## Conclusions

In conclusion, the favorable ICER of the analysis, the confidence in robustness of findings certified by probabilistic sensitivity analysis, the broad coverage rate that GO has achieved in many regions of the Western world, are all positive factors that GO could reinforce its position of valuable asset for the treatment of previously untreated de novo CD33 AML in Italy.

## Supplementary Information

**Additional file 1.**

## Data Availability

The datasets generated and/or analysed during the current study are available from the corresponding author on reasonable request.

## Abbreviations

AIFA: Italian Drug Agency
AML: Acute myeloid leukemia
APL: Acute promyelocytic leukemia
AraC: Cytarabine
BSA: Body Surface Area
CADTH: Canadian Agency for Drugs and Technologies in Health
CR: Complete remission
CRp: Complete remission with incomplete platelet recovery
DA: Daunorubicin and cytarabine
DH: Day-hospital
DNR: Daunorubicin
EFS: Event-free survival
EMA: European Medicines Agency
EQ-5D: European quality of life 5 dimensions
ESMO: European Society for Medical Oncology
FLAG-Ida: Fludarabine, cytarabine, G-CSF and idarubicin
G-CSF: Granulocyte colony stimulating factor
GO: Gemtuzumab ozogamicin
GVHD: Graft versus host disease
HSCT: Hematopoietic stem-cell transplant
HTA: Health Technology Assessment
ICER: Incremental cost-effectiveness ratio
ITT: Intention to treat
IV: Intravenous
KM: Kaplan Mayer curve
LY: Life years
NHS: National Health Service
NICE: National Institute for Health and Clinical Excellence
OS: Overall survival
QALY: Quality adjusted life years
RMST: Restricted mean survival time
SD: Standard deviation
SMC: Scottish Medicines Consortium
UK: United Kingdom
VOD: Veno-occlusive disease
