# Supplementary material for: Cost-effectiveness analysis of gemtuzumab ozogamicin for the treatment of de novo CD33-positive Acute Myeloid Leukaemia (AML) in Italy
Source: BMC Health Serv Res. 2023 Jan 16;23:36. doi: 10.1186/s12913-023-09054-x (PMC9841690; doi:10.1186/s12913-023-09054-x)
Supplement: Supplementary file 1 — Additional file 1. [file 12913_2023_9054_MOESM1_ESM.docx]

# Supplementary materials

**Table 7. Cost inputs**

| **Resource** | **Cost (€)** | **Source** |
| --- | --- | --- |
| Drug acquisition costs | | |
| Chemotherapy | | |
| Mylotarg (5 mg, 1 vial) | 8,500.00^a^ | Ex-factory price [33] |
| Daunorubicin (20 mg, 1 vial) | 8.86 | Ex-factory price, class H [34] |
| Cytarabine (500 mg/5 ml solution, 1 vial) | 6.93 | Ex-factory price, class H [34] |
| Cytarabine (1000 mg/10 ml solution, 1 vial) | 14.33 | Ex-factory price, class H [34] |
| Cytarabine (2000 mg/5 ml solution, 5 vial) | 28.04 | Ex-factory price, class H [34] |
| Fludarabine (50 mg/2 ml concentrato, 1 vial) | 69.26 | Ex-factory price, class H [34] |
| G-CSF (filgrastim) (30 million units/0.5 ml solution, 5 vial) | 95.18 | Retail price, class A [35] |
| G-CSF (filgrastim) (48 million units/0.5 ml solution, 5 vial) | 147.99 | Retail price, class A [35] |
| Idarubicin (5 mg powder for solution, 1 vial) | 51.70 | Ex-factory price, class H [34] |
| Idarubicin (10 mg powder for solution, 1 vial) | 89.93 | Ex-factory price, class H [34] |
| Non-curative therapies | | |
| Cytarabine at low dosage (100 mg, 1 vial) | 1.56 | Ex-factory price, class H [34] |
| Hydroxycarbamide (50.000 mg) | 8.91 | Retail price, class A [35] |
| Azacitidine (25mg/ml 100mg, 1 unit) | 319.49 | Ex-factory price, class H [34] |
| Supportive care | | |
| Posaconazole | 968.17 | Retail price, class A [35] |
| Gentamicin (80mg 2ml, 1 unit parenteral use) | 0.54 | Ex-factory price, class H [34] |
| Gentamicin (240mg, 1 unit parenteral use) | 1.62 | Ex-factory price, class H [34] |
| Gentamicin (360mg, 1 unit parenteral use) | 2.43 | Ex-factory price, class H [34] |
| Veno-occlusive disease treatment | | |
| Defibrotide (200 mg, 1 vial) | 474.09 | Price, class C [22] |
| Costs of drug administration | | |
| Inpatient | | |
| First infusion (day1 of first cycle) | 353.00 | Code: DH 492 [21] |
| Following infusion (cost per infusion) | 353.00 | Code: DH 492 [21] |
| Outpatient | | |
| Oral chemotherapy | 0.00 | *Assumption* |
| First infusion (day1 of first cycle) | 353.00 | Code: DH 492 [21] |
| Following infusion (cost per infusion) | 353.00 | Code: DH 492 [21] |
| Visits | | |
| Patients AML | 669.80 | Average of codes DRG [21] weighed for average days of hospitalization [36]: DRG 473, 492 |
| Hematologic visit | 41.32 | Codes: 89.03, 89.7 [37] |
| Follow-up hematologic visit | 20.66 | Code: 89.7 [13] |
| Specialized nurse (20 minutes) | 0.00 | *Assumption* |
| Blood products | | |
| Red blood cell transfusion | 199.33 | [38] |
| Platelet transfusion | 199.33 | [38] |
| Disease management | | |
| Lab tests | | |
| Blood exam completed | 3.10 | Code: 90.62.2 [37] |
| Biochemical profile | 2.17 | Code: 90.44.3 [37] |
| Liver function test | 2.04 | Codes: 90.04.5, 90.09.2 [37] |
| Blood cultures | 12.19 | Code: 90.86.1 [37] |
| Aspirated bone marrow | 48.86 | Code: 41.31 [37] |
| Bone marrow biopsy of the bone marrow | 48.86 | Code: 41.31 [37] |
| Bone marrow cytogenetics | 33.78 | Code: 91.39.2 [37] |
| Sample extraction | | |
| Blood sample (phlebotomy) | 2.58 | Code: 91.49.2 [37] |
| Bone marrow extraction for diagnostics | 652.00 | Code: DRG 467 [21] |
| Ultrasound examination of the liver (diagnosis of VOD) | | |
| Endoscopic ultrasound examination | 489.00 | Codes: DH 207, 208 [21] |
| Hematopoietic stem cell transplantation | | |
| Average cost per transplant (HSCT) | | |
| Transplant (HSCT) | 81,144.00 | [24] inflated to 2021 [25] |
| From 0 to 6 months post- trasplant (HSCT) | 30,174.50 | *Assumption*: annual follow-up cost of  allo-HSCT, excluding transplant costs [39] |
| From 6 to 12months post- trasplant (HSCT) | 30,174.50 | *Assumption*: annual follow-up cost of  allo-HSCT, excluding transplant costs [39] |
| From 12 to 24months post- trasplant (HSCT) | 1,844.00 | *Assumption*: 1 visit every 3 months (DH 473) [21] |
| Treatment of adverse events | | |
| Skin toxicity | 1,115.50 | Codes: DRG 283, 284 [21] |
| Mucosal toxicity | 1,712.00 | Codes: DRG 182, 183 [21] |
| Ache | 361.90 | [40] inflated to 2021 [25] |
| Nausea, vomiting and diarrhea | 959.00 | Code: DRG 183 [21] |
| Pulmonary toxicity | 3,802.00 | Code: DRG 87 [21] |
| Heart rhythm disorder | 1,674.50 | Codes: DRG 138, 139 [21] |
| Other cardiac toxicity | 2,767.00 | Codes: DRG 124, 125 [21] |
| Central neurological toxicity | 2,723.00 | Codes: DRG 34, 35 [21] |
| Peripheral neurological toxicity | 2,723.00 | Codes: DRG 34, 35 [21] |
| Infections | 3,687.50 | Codes: DRG 79, 80, 320, 321 [21] |
| Hemorrhage | 2,748.00 | Code: DRG 397 [21] |
| Veno-occlusive disease | 12,834.33 | *Calculation*: included [37] and [22] |
| End of life costs | | |
| Last 8 weeks of life | 7,901.40 | [24] inflated to 2021 [25] |

AML = acute myeloid leukemia; DA = daunorubicin and cytarabine; G-CSF = granulocyte colony stimulating factor; GO = gemtuzumab ozogamicin; HSCT = hematopoietic stem-cell transplant; VOD = venu-occlusive disease.

^a^The price used for the analysis is the price at the net of the confidential discounts.
